# Hyperbaric Oxygen Therapy in Traumatic and Non-Traumatic Spinal Cord Injuries: Insights from Nearly Five Decades of Evidence with Single-Center Experience

**DOI:** 10.3390/brainsci16020165

**Published:** 2026-01-30

**Authors:** Giorgio Iaconetta, Carlotta Ranalli, Jacopo Rosso Antonino, Antonio Siglioccolo, Nicola Narciso, Raffaele Scrofani, Ettore Amoroso, Marco Cascella, Matteo De Simone

**Affiliations:** 1 Department of Medicine, Surgery and Dentistry “Scuola Medica Salernitana”, University of Salerno, 84081 Baronissi, Italy; giaconetta@unisa.it; 2 Department of Neurosurgery, Azienda Ospedaliero-Universitaria “San Giovanni di Dio e Ruggi d’Aragona”, University of Salerno, 84131 Salerno, Italy; nicola.narciso@sangiovannieruggi.it (N.N.); raffaele.scrofani@sangiovannieruggi.it (R.S.); ettore.amoroso@sangiovannieruggi.it (E.A.); 3 Department of Medicine and Surgery, Fondazione Policlinico Universitario A. Gemelli IRCCS, 00168 Rome, Italy; carlotta.ranalli01@icatt.it; 4 Department of Biomedical Sciences for Health, Università degli Studi di Milano, 20122 Milan, Italy; jacopoantonino@gmail.com; 5 Department of Intensive Care Unit, Azienda Ospedaliero-Universitaria “San Giovanni di Dio e Ruggi d’Aragona”, 84125 Salerno, Italy; antonio.siglioccolo@sangiovannieruggi.it; 6 Department of Diving and Hyperbaric Medicine, Azienda Ospedaliero-Universitaria “San Giovanni di Dio e Ruggi d’Aragona”, 84125 Salerno, Italy

**Keywords:** traumatic spinal cord injury, non-traumatic spinal cord injury, systematic review, hyperbaric oxygen therapy, HBOT, neuroprotection

## Abstract

**Highlights:**

**What are the main findings?**

- HBOT is associated with neurological improvement in selected patients with a spinal cord injury.
- The treatment response varies according to etiology and timing of intervention.

**What are the implications of the main findings?**

- HBOT may serve as a valuable adjunct in the multidisciplinary management of spinal cord injuries.
- Standardized protocols and controlled trials are needed to define optimal indications and timing.

**Abstract:**

**Background:** Hyperbaric oxygen therapy (HBOT) has been proposed as an adjunctive treatment for spinal cord injuries (SCIs) to mitigate a secondary injury and enhance neurological recovery. While the preclinical evidence is consistently supportive, clinical data remain heterogeneous across traumatic (TSCI) and non-traumatic (NTSCI) etiologies. **Methods**: A hybrid systematic review was conducted in accordance with the PRISMA 2020 guidelines and included an illustrative single-center clinical case. PubMed, OVID Medline, and Google Scholar were searched for studies published between 1978 and 2024. Due to methodological heterogeneity, qualitative synthesis was performed. **Results**: Fifty studies comprising 1102 patients were included. Neurological improvement was more frequently observed in incomplete injuries and when HBOT was initiated early. **Conclusions:** HBOT may represent a useful adjunct in selected SCI patients, although standardized protocols and controlled trials are required to better define its role.

## 1. Introduction

The socioeconomic impact of SCIs is substantial, given their large disability burden and long-term healthcare costs, particularly in young individuals with cervical injuries [1,2,3,4,5,6].

The pathophysiology of an SCI involves a complex and dynamic biphasic process that has been well described in recent years. The primary injury is often the most important determinant of an SCI and is caused by (1) immediate mechanical trauma, (2) compression, (3) shearing, or (4) laceration/stretching, occurring at the time of impact [7]. However, it is the secondary injury cascade that represents both the greatest threat to neurological recovery and the most promising therapeutic target. A secondary injury develops as a result of loss of micro-vascular integrity and decreased cerebral blood flow after contusion and/or compression. This sets off a series of metabolic and pathophysiological events, such as oxidative stress, edema, hypoxia/ischemia, apoptosis, inflammation, and glutamate-mediated excitotoxicity, which together lead to gradual degeneration and the death of neurons [8,9,10]. A secondary injury causes an extensive therapeutic interval that spans from hours to weeks subsequent to the injury, thereby facilitating neuroprotective interventions [11].

Nevertheless, to this date, following numerous years of research, no definitive treatments for SCIs have become accessible. Corticosteroids represent one of the most promising alternatives for managing secondary injuries, although the outcomes of such treatment are challenging to anticipate, and the adverse effects can be considerable [12]. Hyperbaric oxygen therapy (HBOT) has been proposed as a potential adjunctive treatment to mitigate and prevent a secondary injury after an SCI. Specifically, HBOT involves patients inhaling 100% oxygen at a pressure greater than 1.4 atmospheric pressure absolute (ATA), thus increasing their blood oxygen levels to approximately 15-fold higher than those found under normobaric conditions, potentially influencing the pathophysiological processes underlying the secondary injury [13,14]. The biological mechanisms of a secondary injury that HBOT targets are demonstrated in preclinical studies supporting the rationale for HBOT in SCIs. For example, it has been demonstrated that HBOT can raise the dissolved oxygen levels in plasma, which could enhance tissue oxygenation in hypoxic areas of the spinal cord [15,16]. Moreover, it exerts neuroprotection via (i) blood–brain barrier (BBB) stabilization and edema reduction [17], (ii) the regulation of immune responses [18], (iii) the promotion of angiogenesis by VEGF upregulation [19], (iv) the mobilization of the endogenous stem cell system [20], (v) the enhancement of neuroplasticity through neurotrophic factor upregulation [21], and (vi) the protection of mitochondrial function [22]. The preclinical evidence for HBOT in SCIs is substantial and uniformly positive, with animal studies demonstrating reduced lesion size, improved motor functional recovery, enhanced tissue preservation, reductions in inflammation, and amelioration of the electrophysiological outcomes [23,24,25,26,27,28,29,30,31,32]. Although these findings imply that HBOT plays a role in the process of healing, its clinical efficacy in the treatment of SCIs is unclear owing to the disparity of indications and outcomes.

Therefore, the objective of this article is to assess the efficacy of HBOT in enhancing the outcomes and clinical features of patients suffering from TSCIs and NTSCIs, and establish the efficacy of HBOT as compared to a standard treatment or alternative interventions with respect to neurological recovery, motor function, and sensory outcomes for this group of patients.

## 2. Materials and Methods

### 2.1. Study Design

Hyperbaric oxygen therapy was delivered using a hospital-based hyperbaric chamber at the University Hospital of Salerno (Salerno, Italy). Additionally, we conducted a systematic review of the literature to identify previous reports on the use of HBOT in both TSCI and NTSCI management. This review was conducted following the guidelines set forth by the Preferred Reporting Items for Systematic Reviews and Meta-Analyses (PRISMA) 2020 flow diagram [33]. The review protocol was developed a priori, defining the research question using the PICO framework: (P) patients with a spinal cord injury, traumatic or non-traumatic, complete or incomplete; (I) hyperbaric oxygen therapy (HBOT); (C) standard care, sham HBOT, placebo, or other control interventions; (O) motor and sensory function, neurological recovery, quality of life, and functional independence.

An illustrative case is included to contextualize the findings of this systematic review. Its purpose is to exemplify the practical implications of the reviewed evidence and to facilitate interpretation for readers less routinely engaged with this condition.

### 2.2. Search Strategy

A comprehensive literature search was conducted across multiple databases, including PubMed (National Library of Medicine, Bethesda, MD, USA), Google Scholar (Google LLC, Mountain View, CA, USA), and Ovid MEDLINE (Wolters Kluwer, Alphen aan den Rijn, The Netherlands). The search was restricted to the last 60 years. The literature search was performed on 16 June 2025. The search strategy employed a combination of keywords and MeSH terms. Keywords included “spinal cord injury,” “SCI,” “hyperbaric oxygen therapy,” “HBOT,” “secondary injury,” “neurological outcomes”, “case report,” and “case series”. These terms were combined using both “AND” and “OR” Boolean operators to ensure a broad yet focused search (Table 1). To reduce retrieval bias, the PubMed/MEDLINE strategy was expanded to combine MeSH terms with free-text terms searched in title/abstract fields. Specifically, MeSH terms (e.g., “Spinal Cord Injuries”[MeSH], “Hyperbaric Oxygenation”[MeSH]) were combined with free-text synonyms (e.g., spinal cord injury, traumatic, non-traumatic, myelopathy, decompression sickness, HBOT) using Boolean operators. 

### 2.3. Inclusion and Exclusion Criteria

Inclusion criteria were as follows: (1) studies published in English; (2) studies conducted on human participants with either TSCI or NTSCI, with diagnosis supported by clinical features and/or magnetic resonance imaging (MRI); (3) studies evaluating the use of HBOT as a treatment modality; (4) studies comprising case reports, case series, cohort studies (prospective or retrospective), or randomized controlled trials (RCTs); (5) reported neurological, functional, or imaging outcomes related to HBOT, such as ASIA grade, motor or sensory recovery, functional independence, or MRI changes.

Exclusion criteria were as follows: (1) studies involving animal models or in vitro experiments; (2) studies that did not report specific outcomes related to HBOT in the context of SCIs; (3) conference abstracts, editorials, and letters; (4) studies not published in the English language.

### 2.4. Quality Assessment

The methodological quality and risk of bias of the included studies were assessed according to the study design. Randomized controlled trials were appraised using the Cochrane Risk of Bias 2 tool (RoB 2) implemented in Review Manager (RevMan), version 5.4 (The Cochrane Collaboration, London, UK), while non-randomized comparative studies were assessed using ROBINS-I. Cohort studies were additionally evaluated for selection and comparability (Newcastle–Ottawa Scale). Case reports and case series were assessed for reporting quality using Joanna Briggs Institute (JBI) critical appraisal checklists. Quality assessments were used to inform the qualitative interpretation of the findings.

### 2.5. Database Research

**Figure 1:**
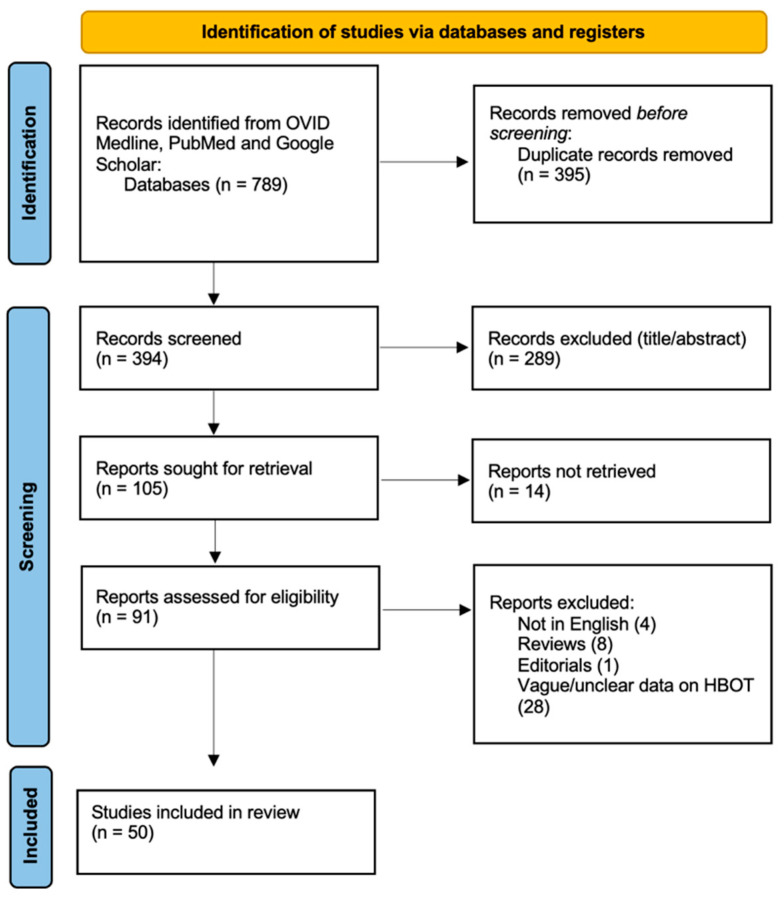
PRISMA flow diagram depicting the literature search process.

A systematic literature review was conducted to assess the available studies on the selected topic. The initial search across three databases (PubMed, OVID Medline, and Google Scholar) identified 789 records. After removing 395 duplicates, 394 records were screened for relevance at the title/abstract level, and 289 records were excluded. Following this screening process, 105 reports were sought for full-text retrieval, but 14 could not be obtained. Of the remaining 91 reports, a further 41 were excluded based on eligibility criteria, including studies not in English (n = 4), reviews (n = 8), editorials (n = 1), and studies presenting vague information on HBOT (n = 28). Ultimately, 50 studies met the eligibility criteria and were included in the final review [34,35,36,37,38,39,40,41,42,43,44,45,46,47,48,49,50,51,52,53,54,55,56,57,58,59,60,61,62,63,64,65,66,67,68,69,70,71,72,73,74,75,76,77,78,79,80,81,82,83] (Figure 1).

### 2.6. Data Synthesis and Analysis

Due to substantial heterogeneity in the study design, patient populations, outcome measures, and HBOT protocols, a quantitative meta-analysis was not feasible. Therefore, the results were synthesized using a qualitative descriptive approach.

## 3. Results

### 3.1. Illustrative Case

**Figure 2:**
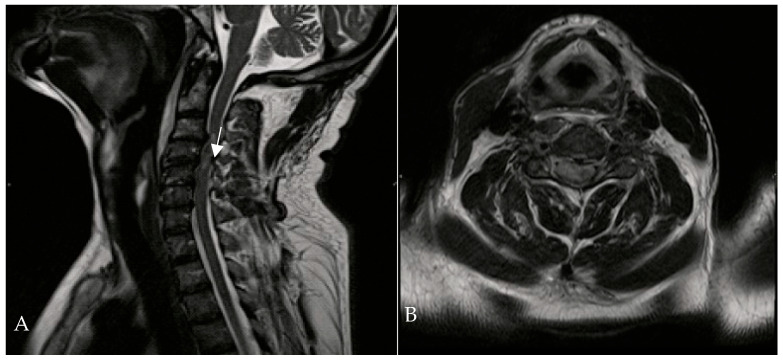
Cervical MRI—T1-weighted sagittal (**A**) and transverse planes (**B**)—showing hematomyelia (white arrow) at the C3-C4 level without vertebral fractures.

A 67-year-old man was admitted to the Reanimation Unit and Neurosurgery Unit of the University Hospital of Salerno, Italy, after a car accident, presenting with dysphonia, urinary and fecal incontinence, and tetraplegia. Despite these severe symptoms, the patient was awake, co-operative, and retained tactile sensitivity in the extremities as well as facial mimicry. Magnetic resonance imaging (MRI) of the spine revealed hematomyelia because of trauma at the C4 level, with no evidence of vertebral fractures. According to the AIS (ASIA Impairment Scale) score, the neurological examination at the time of admission (Time 0) indicated a neurological level of injury (NLI) at C4, classified as AIS B. The patient was initially treated with intravenous corticosteroids (30 mg/kg bolus, followed by 5.4 mg/kg/h infusion over 23 h). On the second day of admission, HBOT was initiated at 1.8 ATA for 60 min per session (Figure 2).

**Figure 3:**
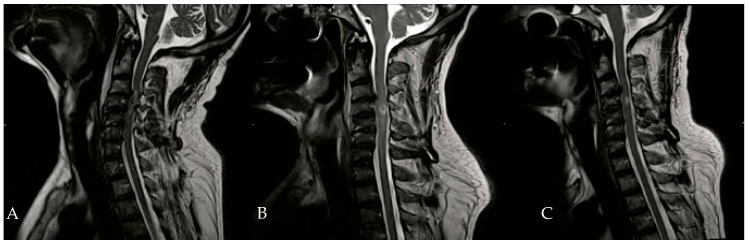
Evolution of hematomyelia in cervical MRI, performed 5 days (**B**) and 30 days (**C**) after the initial MRI (**A**).

In addition to HBOT, the patient received heparin, dexamethasone, omeprazole, and a combination of amoxicillin and clavulanic acid. An Ear, Nose, and Throat (ENT) evaluation revealed a slight adductor deficit of the vocal cords and a minor glottic incompetence due to a superior laryngeal nerve deficit. On the second day of HBOT, hypertonia of the flexor muscles in both hands was noted, prompting the initiation of twice-daily physiotherapy sessions. By the tenth day of HBOT, significant clinical improvements were observed, including the recovery of anal sphincter control, full recovery of neck flexor and extensor muscles, and improved phonation. Subsequent neuroradiological examinations during hospitalization showed a gradual reduction in spinal cord edema (Figure 3).

The patient completed 19 sessions of HBOT, with the neurological level of injury remaining at C4, but improved from AIS B to AIS D. After one month of HBOT, the patient was discharged to a neuro-rehabilitation center for an additional six months of treatment. At the final follow-up, the patient’s neurological examination revealed diffuse spastic hypertonia and a neurogenic bladder. Upper-limb motion showed paralysis in the right-hand flexors and the left elbow and hand extensors, with moderate motor recovery (3/5) in the lower limbs. The patient achieved sufficient recovery to sit and move independently using an electric wheelchair. With regard to autonomic function, no clinically documented episodes of autonomic dysreflexia were observed during the monitored hospital course. Similarly, no clinically significant episodes of orthostatic hypotension requiring specific therapeutic intervention were reported. Bladder and bowel function progressively improved during rehabilitation, in line with the overall neurological recovery.

### 3.2. Non-Traumatic Spinal Cord Injury Patients

Given the marked etiological heterogeneity within NTSCI, and the predominance of decompression sickness, which is pathophysiologically distinct from other non-traumatic causes, NTSCI results are summarized and interpreted by main etiological subgroups (decompression sickness, ischemic/vascular, procedural complications, infectious, radiation-related, and degenerative/structural conditions), with detailed study-level data retained in Table 2 and Table 3.

#### 3.2.1. Demographics, Clinical Presentation, and Initial Treatment: NTSCI Patients

We summarized 25 articles reporting case reports or small series on NTSCI [34,35,36,37,38,39,40,41,42,43,44,45,46,47,48,49,50,51,52,53,54,55,56,57,58]. Across a total of 32 patients, the mean age was 50.6 years (range: 4–75 years). The male-to-female ratio demonstrated a clear male predominance, with 23 males (71.9%) and 9 females (28.1%). The most frequent etiology was radiation-induced NTSCI, accounting for 31.2% (10/32) of cases. This was followed by spinal cord ischemia of vascular origin in 15.6% (5/32), procedural non-vascular complications in 15.6% (5/32), type II decompression sickness in 15.6% (5/32), infection-related NTSCI in 9.4% (3/32), and degenerative or structural causes in 9.4% (3/32). AIS grading was reported in nine patients. The majority presented with complete injuries, classified as AIS A in 66.7% (6/9), including four cases due to type II decompression sickness and two cases related to structural abnormalities or procedural complications. Two patients out of nine were graded as AIS C, both with radiation-induced NTSCI, while one patient with radiation-induced NTSCI was graded as AIS E. The most common initial treatment was supportive care, reported in 34.4% (11/32) of patients. This was followed by corticosteroid therapy in 28.1% (9/32), while no intervention was reported in 18.8% (6/32). Antibiotics were used in 15.6% (5/32), primarily in infectious or radiation-related cases (Table 2).

In addition, we summarized 12 articles that reported the following: one randomized clinical trial (RCT), nine retrospective cohort studies, one prospective cohort study, and one larger case series on NTSCI [59,60,61,62,63,64,65,66,67,68,69,70]. Across a total of 870 patients, the mean age was 50.1 years (range: 29–65.6 years). Similarly to Table 2, here the prevalence was also clearly male-dominated, with 83% of patients being males (714/870) and 17% of patients being females (146/870), resulting in a male-to-female ratio of 4.9:1. The most frequent etiology was decompression sickness, accounting for 72.9% (635/870) of patients. This was followed by procedural non-vascular complications in 15.4% (134/870), infection-related NTSCI in 6.9% (60/870), ischemic/vascular causes in 4.3% (37/870), and degenerative or structural etiologies in 1.1% (10/870). AIS grading was reported only in 12.5% (109/870) of patients. Among these, the majority belonged to the randomized cohort of Chen et al., where 78.5% (73/93) were classified as ASIA E and 21.5% (20/93) as ASIA D [61]. In another series on spinal tuberculosis by Topuz et al., 16 patients were reported as AIS C–D [67]. For the remaining 87.5% (761/870) of patients, AIS data were not specified. In terms of initial treatment, the majority of patients, 76.6% (666/870), received only supportive care. Corticosteroid administration was reported in 10.7% (93/870) of cases, while 1.1% (10/870) started rehabilitation therapy as a primary approach. No specific treatment was provided in 11.6% (101/870) of patients (Table 3).

#### 3.2.2. Imaging, Surgery, and Adjunctive Treatment: NTSCI Patients

In NTSCI case reports and small case series (Table 2), MRI data were available for 90.6% (29/32) of patients, while 9.4% (3/32) had no imaging description reported. The most frequent finding was intramedullary T2 hyperintensity and/or edema, observed in 43.8% (14/32) of cases, often extending over multiple vertebral levels. Cord compression was documented in 28.1% (9/32), most frequently due to infectious or structural causes, including epidural abscesses and osteoradionecrosis with vertebral collapse. Intramedullary enhancement was described in 12.5% (4/32), occasionally associated with diffuse edema, and epidural or extra-axial findings were reported in 6.3% (2/32). Surgical intervention was performed in 35.5% of patients (12/32). The most frequent were decompressive procedures with or without fixation, appearing in 18.6% of patients (6/32), followed by complex reconstructions in 6.3% (2/32) and abscess drainage in 6.3% (2/32) of cases. Most patients did not undergo surgery (18/32), while in two cases the surgical status was not specified. Adjunctive medical therapies were reported in 75% of cases (24/32) following surgery. The most frequent approach was rehabilitation, applied in 25% of patients (8/32), either as a stand-alone treatment or in combination with other agents. Corticosteroids were used in 21.9% of cases (7/32). Antibiotics were administered to 15.6% of patients (5/32), primarily in cases with infectious etiologies, while systemic anticoagulation or heparinization was documented in 6.3% of cases (2/32). No adjunctive medical therapy was recorded in 18% of patients (6/32), and in 6.3% of cases (2/32) details were not specified.

The MRI findings were not included because the majority of the included cohort and trial studies did not systematically report radiological details (Table 3). In contrast to case reports and small case series, where individual imaging findings are often described, larger cohorts summarized clinical outcomes without specifying MRI characteristics. For this reason, the MRI data were considered too heterogeneous and incomplete to be tabulated consistently. Spinal surgery was performed in 22.3% (194/870) of patients. The most frequent procedure was posterior laminectomy and decompression, reported in 10.7% (93/870). Decompression surgery with laminoplasty and fusion was performed in 4.7% (41/870), while debridement with or without fusion was reported in 1.8% (16/870). Surgery for chronic osteomyelitis was undertaken in 5.1% (44/870). The majority of patients, 77.7% (676/870), did not undergo spinal surgery. Finally, adjunctive medical therapies were reported in 38.4% (334/870) of patients. Corticosteroids were by far the most common, administered in 38.3% (333/870) of cases. Antibiotics were used in 5.1% (44/870), mainly for infectious etiologies, while an anti-TB regimen was applied in 1.8% (16/870). CSF drainage with transfusion was reported in 0.8% (7/870). Supportive care was mentioned in 3.4% (30/870). The majority of patients, 61.6% (536/870), did not receive any adjunctive therapy.

#### 3.2.3. HBOT Commencement, Timing, and Sessions: NTSCI Patients

HBOT was most frequently initiated within hours of symptom onset in 46.9% (15/32) of cases, reflecting an emphasis on early treatment (Table 2). A smaller proportion of patients began HBOT immediately after onset in 18.8% (6/32), while delayed initiation was documented in isolated reports, such as post-operative starts in 15.6% (5/32) and markedly delayed initiation at 12 days or even 22 months after radiotherapy (2/32). The number of HBOT sessions varied widely, with most patients receiving between 10 and 30 sessions. The most common regimen was 20 sessions (21.9%, 7/32), followed by 30 sessions (15.6%, 5/32). Outliers included patients receiving fewer than 5 sessions (2/32) or prolonged regimens exceeding 40 sessions (2/32). The treatment pressure (ATA) ranged from 1.8 to 3.0, with the majority of patients treated at 2.0 ATA (43.8%, 14/32). Higher pressures of 2.4–2.5 ATA were used in 21.9% (7/32), while 3.0 ATA was reported in 9.4% (3/32). Session duration was typically 60 to 90 min, with the majority of cases clustering at 90 min (40.6%, 13/32). Longer exposures (≥120 min) were less common, accounting for 12.5% (4/32).

HBOT was most frequently initiated within hours of symptom onset, occurring in 53.1% (462/870). Therapy started within days after the event in 14.1% (123/870), while post-operative initiation was reported in 7.2% (63/870). Delayed initiation occurred in 26.3% (229/870) with a mean of 5 days (range 4 h to 44 days), and 5.1% (44/870) were treated in the chronic phase up to 30 years after the SCI. The number of HBOT sessions ranged widely: the most common regimens included 10–20 sessions, with some protocols reporting 42 sessions or even 30–70 sessions in chronic SCI. ATA varied from 2.0 to 6.0 ATA. Most studies used 2.0–2.8 ATA, whereas higher pressures (≥6.0 ATA) were exclusively applied in decompression sickness cohorts. Session duration was usually 60–90 min, although prolonged exposures up to 330 min were reported in decompression sickness trials (Table 3).

#### 3.2.4. Clinical Outcomes and Follow-Up After HBOT: NTSCI Patients

Clinical outcomes following HBOT in patients with NTSCI (Table 2) were highly variable but overall encouraging. Full neurological recovery was documented in 34.4% (11/32), while partial or moderate improvement occurred in 28.1% (9/32). In contrast, persistent severe deficits despite HBOT were observed in 18.8% (6/32), and progressive neurological worsening was reported in 3.1% (1/32). Stable outcomes without improvement were seen in 15.6% (5/32). Follow-up duration ranged widely, from short-term reassessments at 1–3 weeks to long-term monitoring up to 208 weeks (4 years). Notably, several patients demonstrated delayed but substantial functional gains, including the recovery of continence, restoration of ambulation, and resolution of sensory deficits, often confirmed with concurrent radiological improvement.

Among the NTSCI cohorts and trials (Table 3), full neurological recovery was achieved in 29.5% (257/870), while partial improvement was observed in 44.8% (390/870). Conversely, 21.6% (188/870) had persistent deficits or incomplete recovery, and 4.1% (35/870) experienced treatment failure, recurrence, or no benefit. Follow-up duration was highly variable, ranging from very short-term assessments at 4 weeks to extended monitoring up to 468 weeks (9 years).

### 3.3. Traumatic Spinal Cord Injury Patients

#### 3.3.1. Demographics, Clinical Presentation, and Initial Treatment: TSCI Patients

Four articles reporting case reports or small series on TSCI [71,72,73,74] were summarized (Table 4), including a total of four patients. The mean age was 42.5 years (range: 33–54 years). The male-to-female ratio demonstrated a male predominance, with three males (75%) and one female (25%). The most frequent etiology was Brown-Séquard syndrome, reported in two patients (50%), followed by a traumatic C1 lesion after a diving accident (25%) and a forklift-related lumbar trauma with spondyloptosis (25%).

AIS grading was available in two cases, wherein one patient presented with AIS B and one with AIS C, while in two cases (50%) the AIS score was not specified. The most common initial treatment was pharmacological stabilization (50%, 2/4), consisting of neurotrophic drugs or supportive care, followed by corticosteroids in one case (25%), whereas one patient (25%) did not receive acute medical therapy.

We analyzed nine cohort studies and case series on TSCI [75,76,77,78,79,80,81,82,83], including a total of 232 patients (Table 5). The mean age was 43.3 years (range: 24–60.5 years). Most series reported a clear male predominance, with an overall male-to-female ratio of approximately 3:1, as documented in seven out of nine studies (77.8%). All studies included acute traumatic SCI, with eight cohorts (88.9%, 8/9) focusing on acute cervical or thoracic TSCI and one study specifically addressing cervical hyperextension injuries. In terms of initial treatment, corticosteroids were the most frequently reported, administered in 44.4% (4/9) of studies, covering a total of 136 patients (58.6%). Supportive care alone was adopted in 33.3% (3/9) of studies, accounting for 44 patients over 232. No acute pharmacological or supportive treatment was reported in 22.2% (2/9) of studies, covering 52 patients over 232. Neurological severity was heterogeneous, ranging from AIS A to D across the included series. In Zhang et al. (2023), 100% (40/40) of patients were AIS B–D at admission, while Tan et al. (2023) reported AIS A–D distribution in 29 patients [71,72].

#### 3.3.2. Imaging, Surgery, and Adjunctive Treatment: TSCI Patients

In Table 4, spinal surgery was performed in three patients (75%), including posterior decompression with fixation at C1-C2, posterior instrumentation with cage fusion at L3-S2, and decompressive laminectomy with fragment removal at T5. Only one patient (25%) did not undergo surgical treatment. Adjunctive therapies were reported in three patients (75%), most frequently rehabilitation (with or without acupuncture), while antibiotics were administered in one case (25%).

In Table 5, surgical intervention was reported in 55.6% (5/9 studies), involving 40.9% (95/232) of patients, most commonly early decompression and fixation procedures. The remaining 59.1% (137/232) were managed conservatively. Regarding adjunctive therapies, rehabilitation was administered in 43.5% (101/232) of patients, corticosteroids in 13.8% (32/232), and other adjunctive measures such as mannitol, mecobalamin, or acupuncture in 8.7% (20/232).

#### 3.3.3. HBOT Commencement, Timing, and Sessions: TSCI Patients

In Table 4, HBOT was typically initiated early during hospitalization or within 10 days after injury in two patients (50%), while in one patient, treatment was started 3 months post injury, and in another case, the timing was not specified. The number of sessions varied widely, ranging from 60 sessions to 127 sessions, with pressures of 2.0–2.5 ATA and session durations of 60–90 min. This large variation reflects a tendency in small series toward prolonged or individualized regimens, often used in the subacute or chronic rehabilitation phase. In contrast, Table 5 shows more standardized treatment protocols: HBOT was initiated within hours of injury in 30.2% (70/232) and within 3–15 days post operation in 38.8% (90/232), while late initiation, ≥90 days, was reported only in one cohort (40/232). Many patients underwent 10–20 sessions, with extended regimens of 30–48 sessions in selected series. Pressures were generally 2.0–2.5 ATA in 7/9 of studies, with occasional higher values up to 3.0 ATA, and session durations were most frequently 60–90 min, with some prolonged up to 160 min.

#### 3.3.4. Clinical Outcomes and Follow-Up After HBOT: TSCI Patients

Clinical outcomes were favorable in most cases (Table 4). One patient improved from AIS B to AIS D, regaining partial independence with aids at 78 weeks; another improved from AIS C to AIS D with pain relief and resolution of urinary retention at 24 weeks. One patient with a cervical diving injury demonstrated significant improvement in proprioception and motor performance after 127 sessions, while the fourth patient achieved near-complete motor recovery one year after decompressive laminectomy and HBOT.

Across the nine cohort studies and case series on TSCI, outcomes following HBOT were variable but generally favorable, with the majority of patients demonstrating neurological improvement (Table 5). Overall, seven of nine studies reported measurable AIS grade improvements, most frequently from incomplete injuries (AIS B–D) toward functional recovery (AIS C–E). Early HBOT response was associated with better prognosis, as observed in Ishihara et al., where most patients improved to AIS D/E at long-term follow-up [79]. In the largest retrospective series by Zhang et al. [75], sustained recovery to AIS C–E was noted at 156 weeks, while the recent trials by Sun et al., Tan et al., and Feng et al. confirmed AIS improvement within shorter follow-up intervals of 4–8 weeks, with Tan et al. documenting 86.2% of patients achieving ≥1 AIS grade improvement and concomitant MRI lesion size reduction [76,77,78]. Outcomes in older series were more heterogeneous: Asamoto et al. reported recovery from AIS A/B to E in hyperextension injuries [80], while Gamache et al. found that only 4/25 incomplete SCIs improved, with complete injuries showing no recovery at 24 weeks [81]. Similarly, Jones et al. and Yeo et al. demonstrated partial neurological improvement in 50–70% of patients, though mortality was reported in one case [82,83].

**Figure 4:**
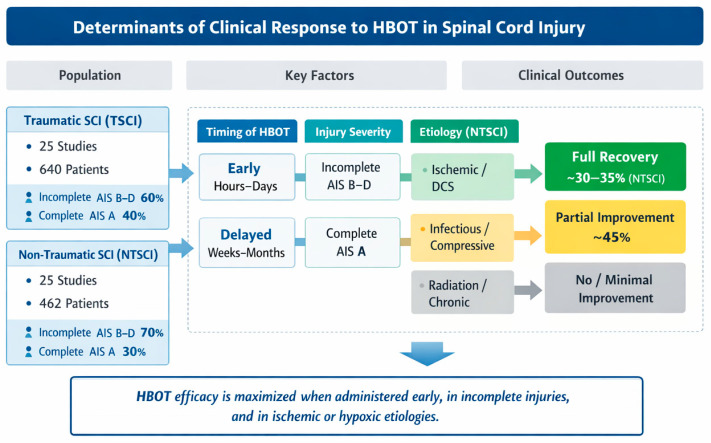
Conceptual summary of factors influencing clinical responses to HBOT in TSCI and NTSCI patients. The dashed boxes (A–D) represent the main determinant domains analyzed across the included studies: (A) timing of HBOT initiation (early vs. delayed), (B) neurological injury severity according to the ASIA Impairment Scale, (C) injury etiology, and (D) corresponding clinical outcomes.

Figure 4 provides a graphical synthesis of the main findings, highlighting the key determinants influencing clinical responses to hyperbaric oxygen therapy. The figure integrates patient characteristics, injury-related factors, and treatment timing to facilitate the interpretation of outcome patterns across studies.

## 4. Discussion

One limitation in interpreting the observed clinical improvements is the frequent concomitant use of standard therapies, including corticosteroids, anticoagulation, surgical decompression, and structured rehabilitation. These interventions are known to influence neurological recovery after SCI and may act as confounding factors. Accordingly, HBOT should be regarded as an adjunctive treatment whose potential benefits likely derive from synergistic effects with standard care rather than from an isolated therapeutic action.

This article provides the most comprehensive synthesis to date of the clinical evidence on hyperbaric oxygen therapy for spinal cord injury, encompassing both traumatic and non-traumatic etiologies. While experimental data have consistently demonstrated HBOT’s potential to attenuate secondary injury cascades, clinical translation remains heterogeneous, reflecting differences in the timing of initiation, treatment protocols, and patient populations.

For both TSCIs and NTSCIs, HBOT has been investigated as an adjunctive treatment aimed at limiting secondary damage and enhancing neurological recovery.

### 4.1. Clinical Evidence in Traumatic Spinal Cord Injury Patients

For TSCI cases, our compiled data (Table 4 and Table 5) indicate that HBOT is typically initiated in the acute phase, often within hours or days post injury or post surgery, in conjunction with standard interventions like spinal decompression, high-dose steroids, or intensive rehabilitation. Across multiple studies, TSCI patients treated with HBOT have shown meaningful neurological improvements, especially when it is an incomplete, AIS grade B–D injury. For example, Tan et al. reported that 86.2% of the patients receiving HBOT achieved a one-grade improvement on the ASIA Impairment Scale, along with radiological evidence of reduced lesion size on MRI [77]. Similarly, a randomized trial by Sun et al. found greater ASIA score gains in the HBOT group compared to controls [76]. In general, patients with residual distal function at baseline tend to benefit the most: several studies noted significant motor and sensory gains in incomplete injuries, whereas complete (AIS A) injuries showed minimal to no recovery. This dichotomy was exemplified in an early cohort by Gamache et al., where 4 of 25 incompletely paralyzed patients improved substantially with HBOT, yet none of the complete paraplegics recovered function [81]. Demographically, the TSCI cohorts were predominantly male, as for the institutional case, and relatively young adults, with a mean age between 30 and 50 years, reflecting the typical epidemiology of TSCI [1].

HBOT protocols in TSCI varied, but most employed 100% oxygen at 2.0 ATA for 60–120 min per session. The number of sessions ranged widely: modern protocols often provide 30 daily sessions, whereas some early case series delivered only a few high-pressure sessions in the first 1–2 days post injury.

Notably, earlier HBOT appears to be correlated with better outcomes: Ishihara et al. observed that patients who responded to an initial HBOT session, given within the first day or two after surgery, went on to achieve greater recovery, with most improving to nearly normal motor function (AIS D or E) by final follow-up [79].

Likewise, Asamoto et al. initiated HBOT within hours of a cervical hyperextension injury and reported that even some initially complete lesions improved, with AIS A cases converting to B and B/C to E over the ensuing weeks [80]. These findings underscore a potential time-dependent neuroprotective effect of HBOT in acute TSCI. Experimental studies investigating the neuroprotective effects of HBOT in spinal cord injury have demonstrated that the therapy significantly attenuates the SCI-induced overproduction of pro-inflammatory cytokines, including interleukin-1β (IL-1β) and tumor necrosis factor-α (TNF-α). Concurrently, HBOT has been shown to increase the number of cells positive for glial-cell-line-derived neurotrophic factor and vascular endothelial growth factor (VEGF), as well as to enhance spinal cord IL-10 production, thereby promoting an anti-inflammatory and neuroprotective microenvironment [84]. Moreover, according to Yang et al. [85], HBOT reduces spinal cord edema, enhances neuronal function, and preserves blood–spinal cord barrier integrity by suppressing the expression of matrix metalloproteinase (MMP)-2, interleukin-6 (IL-6), and MMP-9, while promoting vascular endothelial growth factor (VEGF) expression.

Even subacute/chronic TSCI cases, though less responsive, may derive some benefit: one report documented a patient treated with HBOT 3 months after a cervical injury who showed notable gains in proprioception and motor function, accompanied by evidence of cortical connectivity reorganization on follow-up imaging [73]. This suggests that HBOT might facilitate neuroplastic changes, though such dramatic late improvements are rare. Several pieces of evidence support this statement: Chen et al. demonstrated that HBOT prevented cognitive decline in a D-galactose-induced aging mouse model by reducing oxidative stress and inhibiting activation of the NF-κB signaling pathway [86]. Additional studies have shown that HBOT may mitigate Alzheimer’s disease-related pathologies by attenuating neuro-inflammatory processes, as reflected by reduced microgliosis and astrogliosis, decreased levels of TNF-α and IL-1β, and the upregulation of anti-inflammatory and reparative markers, including scavenger receptor A, arginase-1, IL-4, and IL-10 [87]. Similarly, experimental research in Parkinson’s disease models has reported that prolonged exposure to mild hyperbaric oxygen preserves dopaminergic neurons in toxin-induced parkinsonism, supporting a broader neuroprotective role of HBOT [88]. In this context, the illustrative case presented herein exemplifies how these mechanistic insights may translate into clinical practice, providing a concrete representation of the therapeutic effects discussed above.

### 4.2. Clinical Evidence in Non-Traumatic Spinal Cord Injury (NTSCI) Patients

In NTSCI contexts (Table 2 and Table 3), HBOT has been applied to a heterogeneous range of etiologies, including spinal cord ischemia from vascular events or surgery, decompression sickness, radiation myelopathy, neoplastic cord compression, and infectious myelopathies. Overall, the data show generally favorable outcomes in many NTSCI cases, though success varies by cause and timing. In acute ischemic myelopathies, HBOT can be pivotal; for instance, in spinal cord infarction or ischemia, early HBOT has been associated with significant neurological recovery. One Canadian series of 30 patients with paraplegia from post-operative spinal ischemia reported that 56.7% improved, and 26.7% achieved full recovery, after receiving 1–11 HBOT sessions, initiated as soon as possible after the insult [59]. Another study of seven patients with aortic surgery-related SCI likewise noted that five of seven had substantial neurological improvement when HBOT was started within 8–30 h of onset [62]. Spinal decompression sickness (DCS) is a well-established indication for HBOT, and the outcomes in our review reflect its efficacy. Moreover, in a French retrospective cohort of 102 divers with type II spinal DCS, one-third of patients made full neurologic recoveries and nearly half showed partial improvement after prompt HBOT, with only 21% remaining neurologically impaired [60]. Larger analyses of DCS cases similarly show that a substantial fraction can regain normal or near-normal function if treated, though delays in therapy reduce the likelihood of complete recovery [68]. These vascular NTSCI scenarios highlight the critical importance of rapid HBOT to restore spinal cord oxygenation. For both spinal infarction and decompression sickness, treatment is typically initiated within hours of symptom onset, reflecting the same urgency emphasized in TSCI management.

Outcomes in non-traumatic, non-ischemic myelopathies are more variable. In radiation-induced myelopathy, HBOT aims to revascularize and halt the progression of radiation damage, but the evidence is mixed. Calabrò et al. reported a case of cervical radiation myelitis where 20 HBOT sessions led to clear neurological improvement and MRI changes, with the resolution of cord edema and reductions in enhancing lesions [58]. In contrast, a small series by Bünül et al. found no clinical improvement with HBOT in delayed radiation myelopathy, suggesting that established radiation necrosis may be difficult to reverse, or that earlier or more prolonged treatment might be required for effect [38]. For infectious and inflammatory causes, HBOT primarily serves to improve tissue healing and infection control, with neurological benefits being secondary. For example, HBOT has been successfully used to treat spinal osteomyelitis in chronic SCI patients: in one cohort, adding HBOT at 2.0 ATA for 120 min per session to surgery and antibiotics cured 68% of chronic spinal osteomyelitis cases and reduced recurrence rates [70]. In spinal tuberculosis complicated by paraplegia, a study of 16 patients noted that adjunctive HBOT, delivered as 42 sessions at 2.4 ATA combined with standard anti-TB therapy, led to improvements in neurologic status, with most patients improving to AIS D or E and experiencing relief of pain [67]. There is also evidence that HBOT can aid recovery in certain degenerative or compressive myelopathies. Tofuku et al. described 10 patients with cervical spondylotic amyotrophy, a motor deficit syndrome caused by chronic compression, who underwent HBOT during rehabilitation; significant gains in upper-extremity strength were observed, implying that HBOT helped to maximize their neurological recovery even months after symptom onset [65]. Another notable example comes from an older Japanese study by Ishihara et al. on cervical spondylotic myelopathy: a single pre-operative HBOT session was used as a provocative test of tissue viability, and indeed 78% of patients showed excellent post-operative neurologic outcomes when this protocol was applied, hinting that HBOT may synergize with surgical decompression in certain chronic compressive lesions [69]. For instance, DCS requires high-pressure, long-duration treatments at 2.8 ATA for up to 4–5 h per session, whereas infectious or radiation-related cases may use more standard pressures at 2.0–2.4 ATA for about 90 min but a greater number of sessions, typically 20–40 or more, to promote tissue repair.

### 4.3. Comparison of TSCI and NTSCI Findings

When comparing TSCI vs. NTSCI, several distinctions and parallels emerge (Table 6). The timing of HBOT administration is critical in both groups, as the best outcomes are consistently associated with early treatment, capitalizing on a window of opportunity to mitigate secondary injury. In acute trauma, acute ischemia, or DCS, HBOT within the first hours to days can make the difference between partial recovery and permanent deficit. Both TSCI and vascular NTSCI protocols emphasize this urgency: many TSCI studies started HBOT by post-operative day 0–3 or even within hours of injury, and NTSCI guidelines for DCS/spinal stroke similarly advocate for immediate intervention. By contrast, for chronic NTSCI, such as in cases of late radiation myelopathy or long-standing compression, delayed HBOT tends to yield modest improvements at best, highlighting that HBOT I have checked and revised allis not a cure for established irreversible damage but rather a means to rescue compromised but viable tissue.

Another difference lies in treatment strategies and adjunctive care: TSCI patients uniformly receive trauma care, which includes surgical stabilization when needed, anti-inflammatory drugs such as methylprednisolone, and structured rehabilitation, alongside HBOT. For NTSCI, management is etiology-specific for example, an ischemic myelopathy patient might get blood pressure support or a CSF drain to improve spinal cord perfusion, an infectious case will get antimicrobials and possibly surgical debridement, and a DCS patient requires rehydration and possibly additional recompression treatments. Notably, corticosteroids are a common adjunct in TSCI and some NTSCI cases, where they are administered for acute trauma as well as in cases of acute ischemia or DCS to reduce cord swelling.

Patient profiles also differ: TSCI cohorts are skewed toward young males due to the nature of the trauma, whereas NTSCI spans a broader age range depending on the cause, with older patients typically affected in degenerative or vascular cases and younger individuals in DCS cases. Despite these differences, the outcomes reported share a key theme: for both TSCIs and NTSCIs, when HBOT is effective, it often translates to improved motor scores, sensory function, and independence in activities of daily living. Furthermore, both domains report the safety and tolerability of HBOT: none of the studies in our tables identified serious HBOT-related adverse events, echoing the wider literature in evidencing that HBOT is a safe adjunct for SCI patients.

Table 6 summarizes the main differences between traumatic and non-traumatic spinal cord injuries, starting from etiology and extending to the response timing to HBOT, pattern of clinical responses, predominant mechanisms of efficacy, and strength of the supporting evidence.

## 5. Limitations

This study has several limitations. First, many of the included reports are case series or small retrospective cohorts, with only a few randomized or prospective studies available. This design imbalance increases the risk of selection and publication bias, as patients who responded favorably to HBOT may be disproportionately represented. Second, there is marked heterogeneity in the treatment protocols, with substantial variability in the timing of HBOT initiation, number of sessions, and pressure regimens, which limits our ability to draw firm conclusions regarding optimal therapeutic parameters. Another limitation lies in the inconsistency of outcome reporting. While some studies document ASIA grades or detailed functional measures, others rely on narrative clinical descriptions or radiological improvement, preventing a uniform assessment of neurological recovery. Imaging data were also incompletely reported in larger cohorts, reducing our ability to correlate radiological changes with functional outcomes. Finally, the overall sample size, though larger than in previous reviews, remains relatively small when stratified by etiology, particularly for less common NTSCI subgroups such as radiation myelopathy or chronic infections. As a result, the findings should be interpreted as exploratory and hypothesis-generating, rather than definitive. Despite these limitations, our review synthesizes more than four decades of clinical evidence, distinguishes between traumatic and non-traumatic SCI populations, and highlights consistent patterns of benefit, particularly regarding the greater responsiveness of incomplete injuries and the importance of early HBOT initiation. These observations provide a valuable framework for refining future research and guiding the design of larger, multi-center randomized trials to establish the true role of HBOT for SCI.

## 6. Conclusions

HBOT shows promise as an adjunctive therapy for spinal cord injury. In cases of TSCI, it is most effective in patients with incomplete lesions and when initiated early, with evidence of neurological and radiological improvements. In cases of NTSCI, outcomes are favorable in patients with ischemic etiologies and decompression sickness, but more variable in radiation-induced and chronic cases. Protocols remain heterogeneous, and most studies are small. Larger randomized trials with standardized outcome measures are needed to define efficacy, optimize regimens, and identify the patients most likely to benefit.

## Figures and Tables

**Table 1:** Search terms used in electronic databases.

Database	MeSH and Search Terms
MEDLINE/PubMed	**MESH:** “Spinal Cord Injuries”[MeSH]; “Spinal Cord Diseases”[MeSH]; “Spinal Cord Ischemia”[MeSH]; “Myelitis”[MeSH]; “Paraplegia”[MeSH]; “Quadriplegia”[MeSH]; “Hyperbaric Oxygenation”[MeSH]; “Decompression Sickness”[MeSH]; “Tuberculosis, Spinal”[MeSH]; “Osteoradionecrosis”[MeSH]; “Hematoma, Epidural, Spinal”[MeSH]; “Abscess”[MeSH]; “Treatment Outcome”[MeSH]; “Recovery of Function”[MeSH]; “Prognosis”[MeSH]
	**Search Terms**: ((“spinal cord injury”[Title] OR “spinal cord ischemia”[Title] OR “traumatic spinal cord injury”[Title] OR “non-traumatic spinal cord injury”[Title] OR “radiation myelopathy”[Title] OR “Brown-Séquard”[Title]) AND (“hyperbaric oxygen therapy”[Title] OR “HBOT”[Title] OR “HBO therapy”[Title] OR “hyperbaric oxygenation”[Title])) AND (“neurological recovery”[Title] OR “functional outcome”[Title] OR “AIS”[Title] OR “ASIA score”[Title] OR “motor recovery”[Title] OR “sensory recovery”[Title] OR “case report”[Title] OR “case series”[Title])

**Table 2:** NTSCI case reports/small series.

Author, Year, Coun try	Age, Sex	Etiology (Macrocategory)	Specific Condition	Initial Treatment	AIS Grade	MRI	Spinal Surgery	Adjunctive Medical Therapy	Start of HBOT	N° of Sessions	ATA	Duration of Each Session (min)	Outcome	Follow-Up (Week)
Brenna et al., 2023, Canada [34]	50, F	Procedural (direct/non-vascular)	Neurological injury after complex spinal surgery	None	NS	NS	Decompression L2-S1, PLIF L4-S1, osteotomy L2-L4, fusion T5-sacrum	Rehab	Post-op day 4	12	2.0	90	Marked neurological recovery, near-complete at 2 years	104
Liu et al., 2023, China [35]	24, M	Degenerative/structural	Thoracic spinal stenosis from DISH and Scheuermann’s disease	Corticosteroids	NS	Cord compression (thoracic stenosis T7-T9, dural sac compression with vertebral wedging T8-T12)	Thoracic laminectomy + fixation	Electrical stimulation, rehab	Post op (day NS)	NS	NS	NS	Pain resolved, sensation normal, able to walk 1.5 km	NS
Romano et al., 2022, Portugal [36]	32, F	Procedural (direct/non-vascular)	Pneumorrhachis with paraparesis after epidural analgesia	Supportive care	NS	Epidural air (L2-L3)	None	NS	8 h after symptom onset	1	2.8	145	Full neurological recovery	1
Maroon et al., 2021, USA [37]	25, M	Degenerative/structural	Axial load injury with idiopathic scoliosis	Corticosteroid	AIS A	Intramedullary T2 hyperintensity (T8) + cord compression from hematoma (T9)	Decompression + fixation T8-T10	Omega-3 FA, rehab	Post-op day 2	30	2.4	90	Improved to AIS D; able to run and jump at 18 months, mild residual spasticity	78
Bünül et al., 2021, Turkey [38]	70, M	Radiation-induced	Radiation myelopathy	Corticosteroids	NS	Intramedullary T2 hyperintensity (T5)	None	Rehab	12 days after symptom onset	NS	NS	NS	No neurologic improvement	52
	44, F					Intramedullary T2 hyperintensity (T7-9)								
	47, M					Intramedullary T2 hyperintensity (T9-L1)								
Cheng et al., 2021, UK [39]	38, M	Decompression sickness	Type II decompression sickness	Supportive care	ASIA A	Intramedullary T2 hyperintensity (diffuse, upper cervical to entire cord)	None	Rehab	Within hours of symptoms	26	NS	NS	Persistent T9 paraplegia	8
Yin et al., 2019, China [40]	35, M	Procedural (direct/non-vascular)	Intradural hematoma after spinal anesthesia (unrecognized spina bifida + tethered cord)	Corticosteroids	ASIA A	Cord compression (intradural mass, T2 hypointense at T12-S2)	T12-S1 laminectomy, hematoma evacuation, pedicle fixation	Rehab	Post op (day NS)	NS	NS	NS	Improved to AIS C	24
Saadi et al., 2019, USA [41]	65, M	Decompression sickness	Type II decompression sickness	Supportive care	NS	NS	NS	Corticosteroids	Within 6 h from onset	20	2.0	120	Improved to independent walking and continence	48
West et al., 2019, USA [42]	42, M	Degenerative/structural	Craniocervical osteoradionecrosis	Supportive care	NS	Structural lesion with cord compression (clivus-C2 destruction, basilar invagination)	Posterior occipito–cervical fusion (Occ–C7)	None	Pre op and post op (day NS)	40 (20 pre op, 20 post op)	2.5	90	Solid fusion, pain resolution, return to work	104
	63, M							Antibiotics						52
Sahin et al., 2019, Turkey [43]	4, M	Infectious	Intradural spinal cord abscess after prior surgery for spina bifida + tethered cord	Antibiotics	NS	Cord compression (intradural abscess, T10-S2)	Abscess drainage	Antibiotics, corticosteroids	After antibiotic failure	20	2.5	120	Full neurological recovery, walking independently; MRI resolution	5
McClelland et al., 2018, USA [44]	55, F	Radiation-induced	Radiation myelopathy	Corticosteroids	NS	Intramedullary enhancement (T4-T5) with edema extending to C6-C7	T3-T5 posterior decompression + fusion	Corticosteroids	Post op (day NS)	NS	NS	NS	Moderate sensory improvement	NS
Rashid et al., 2017, Malaysia [45]	64, F	Radiation-induced	Sub-axial cervical osteoradionecrosis	Supportive care	NS	Structural lesion/osteoradionecrosis with vertebral instability (C3-C5 anterolisthesis, vacuum cleft sign at C4)	Anterior C-spine discectomy ×3, posterior fusion	None	Post-op day 3	17	NS	NS	Full neurological recovery	52
Urquieta et al., 2017, USA [46]	73, M	Ischemic/vascular	Spinal cord ischemia after endovascular aneurysmal repair	CSF drainage	NS	NS	None	None	Within 5 h from onset	2	2.0	90	Full neurological recovery	36
Nozaki et al., 2017, Japan [47]	61, F	Radiation-induced	Radiation myelopathy	Corticosteroids	NS	Intramedullary enhancement (T7-T9)	None	Corticosteroids, heparin	22 months after RT	10	2.0	60	Progressive worsening to complete paraplegia within 3 months	40
Xu et al., 2016, China [48]	45, M	Procedural (direct/non-vascular)	Acute spinal cord ischemia after CT-guided lung biopsy	Supportive care	NS	Intramedullary T2 hyperintensity with cord swelling (T7-T9)	None	Rehab	Within hours of onset	NS	NS	NS	Partial recovery: able to stand 10 min, independent defecation, wheelchair dependent	24
Nishioka et al., 2016, Japan [49]	63, F	Ischemic/vascular	Spinal cord ischemia after endovascular aneurism repair	None	NS	Intramedullary T2 hyperintensity (conus medullaris)	NS	None	Post-op day 3	10	2.0	90	Near-complete resolution of bladder/rectal incontinence	3
Yang et al., 2016, China [50]	55, F	Procedural (direct/non-vascular)	Acute paraplegia after epidural angiomatous meningioma removal	None	NS	Epidural lesion (T6-T8)	T6-T8 laminectomy + en bloc tumor removal	Corticosteroids, rehab	Within hours of onset	NS	NS	NS	Total recovery	24
Morishita et al., 2014, Japan [51]	44, M	Ischemic/vascular	Paraplegia after acute type B aortic dissection	None	NS	Intramedullary T2 hyperintensity (T12)	None	Systemic heparinization	Immediately after onset	NS	2.0	NS	Full recovery within 24 h	12
Ueki et al., 2014, Japan [52]	68, M	Infectious	Cervical spine osteomyelitis + epidural abscess	Antibiotics	NS	Cord compression (osteomyelitis C4-C7 with epidural abscess)	None	Antibiotics	After progression on antibiotics	25	NS		Neurological improvement; reduction of abscess	12
Lee et al., 2010, USA [53]	58, M	Ischemic/vascular	Acute spinal cord ischemia after vertebral artery embolization	None	NS	Restricted diffusion (C2-C5)	None	Systemic anticoagulation, hypothermia	Within hours of onset	5	2.0	90	Sensory loss resolved; walking independently at 3 weeks, near-complete recovery	12
Tofuku et al., 2008, Japan [54]	75, F	Ischemic/vascular	Spinal cord infarction after endoscopic variceal ligation	Supportive care	NS	Intramedullary T2 hyperintensity (C6-T5)	None	None	Immediately after onset	20	2.0	60	Near-complete neurological recovery	12
Yoshiyama et al., 2007, Japan [55]	39, M	Decompression sickness	Type II decompression sickness	Supportive care	AIS A	Intramedullary T2 hyperintensity (T3)	None	Corticosteroids, rehab	Within hours of onset	8	1.8	NS	Improved to AIS D at 6 months	24
	43, M					Intramedullary T2 hyperintensity (C7-T1)				16	3.0		Improved to AIS D at 6 months	
	48, M					Intramedullary T2 hyperintensity (T6-T9)				12	2.0		Minimal improvement to AIS B	
Donovan et al., 2005, USA [56]	62, M	Radiation-induced	Cervical osteoradionecrosis C5-C7 + epidural abscess with cord compression	Antibiotics	AIS C	Cord compression (vertebral body destruction with epidural abscess)	Corpectomy + fibular graft + fixation	Antibiotics	Post op (day NS)	30	2.4	90	Full neurological recovery	104
	69, M		Cervical osteoradionecrosis C1-C2 + epidural abscess with cord compression			Cord compression (C1-C2 bone destruction with epidural abscess)								208
	71, M		Cervical osteoradionecrosis without cord compression	None	AIS E	Osteoradionecrosis	None	None	Immediately after onset				Pain and dysphagia improved; MRI lesion reduced	52
Kohshi et al., 2005, Japan [57]	49, M	Infectious	Cervical spinal epidural abscess with neurological deficit	Antibiotics	NS	Cord compression (epidural abscess C1-C4)	None	Antibiotics	After failure of antibiotics	30	2.5	60	Neurological deficits resolved by day 13; MRI normalized by week 7	24
Calabrò et al., 2000, Italy/USA [58]	71, M	Radiation-induced	Radiation-induced myelopathy	Corticosteroids	NS	Intramedullary enhancement (C3-C4) with diffuse edema (C2-T3)	NS	Corticosteroid vitamins	At symptom onset	20	2.4	90	Neurological improvement: edema resolved; enhancing lesions reduced	20

**Table 3:** NTSCI cohort studies/trials.

Author, Year, Country	N° of Patients (HBOT cohort)	Sex	Mean Age	Study Type	Etiology (Macrocategory)	Specific Condition	Initial Treatment	AIS Grade	Spinal Surgery	Adjunctive Medical Therapy	Start of HBOT	N° of Sessions	ATA	Duration of Each Session	Outcomes	Follow-Up
Lee et al., 2024, Canada [59]	30	22 M/8 F	65.6	Retrospective case series	Ischemic/vascular	Spinal ischemia after complex aortic repair	Supportive care	NS	None	Supportive care	Within hours of onset	1–11	2.0	90	56.7% improved, 26.7% full recovery; 36.7% no improvement; mean motor function gain +16.6 in improvement group	4–24
Simonnet et al., 2023, France [60]	102	82 M/20 F	52	Retrospective cohort	Decompression sickness	Type II decompression sickness	Supportive care	NS	None	Corticosteroids	Within hours of onset	1–15	2.0–2.8	290	33% full recovery, 46% partial, 21% persistent severe deficits	4–24
Chen et al., 2019, China [61]	93	54 M/39 F	47.1	Randomized cohort	Procedural (direct/non-vascular)	Cervical/thoracic spinal stenosis at risk for spinal cord ischemia-reperfusion injury (SCIRI) caused by surgical decompression	Corticosteroids	ASIA E (78.5%), ASIA D (21.5%)	Posterior laminectomy decompression	Corticosteroids	1 week pre op, post-op day 3	14 (7 pre op, 7 post op)	2.0	60	All recovered within 2 weeks	2
Parotto et al., 2018, Canada [62]	7	6 M/1 F	56.5	Retrospective cohort	Ischemic/vascular	Spinal cord ischemia after complex aortic repair	Supportive care	NS	None	CSF drain, transfusion	8–30 h after onset	1–11	2.0–2.8	NS	5/7 neurological recovery	4–24
Chung et. al, 2017, Korea [63]	12	10 M/2 F	39.1	Retrospective cohort	Decompression sickness	Type II decompression sickness	Supportive care	NS	None	Corticosteroids	Within hours of onset	3–15	2.0–2.8	290	5 full recovery; 7 residual weakness/voiding difficulty	4–12
Gao et al., 2014, China [64]	7	7 M	35	Retrospective case series	Decompression sickness	Type II decompression sickness	Supportive care	NS	None	None	Within 1–6 days after symptom onset	4–16	2.5	90	Partial to good neurological recovery	24
Tofuku et al., 2011, Japan [65]	10	7 M/3 F	58	Case series	Degenerative/structural	Cervical spondylotic amyotrophy	Rehab	NS	None	None	Mean 3.1 months after symptom onset	10–20	2.0	60	Significant neurological recovery: improvement of upper-limb weakness	52–156
Blatteau et al., 2011, France/Belgium [66]	279	226 M/ 53 F	42	Retrospective cohort	Decompression sickness	Type II decompression sickness	Supportive care	NS	None	None	Within hours of onset	NS	2.8–6.0	NS	26% incomplete recovery at 1 month	4
Topuz et al., 2009, Turkey [67]	16	10 M/6 F	29	Retrospective cohort	Infectious	Spinal tuberculosis (Pott’s disease with paraplegia/paraparesis)	None	AIS C–D	Debridement ± fusion	Anti-TB regimen	Post op (day NS)	42	2.4	90	Improvement to AIS D–E; pain resolution	260
Barratt et al., 2004, Honduras/Nicaragua [68]	229	229 M	29	Retrospective cohort	Decompression sickness	Type II decompression sickness	Supportive care	NS	None	Corticosteroids	Delayed, mean 5 d (range 4 h–44 d)	NS	2.8	120–330	69/229 patients regained normal strength; 46/229 patients had persistent deficits	
Ishihara et al., 1997, Japan [69]	41	27 M/14 F	60	Prospective cohort	Procedural (direct/non-vascular)	Cervical compression myelopathy	None	NS	Decompression surgery (laminoplasty, fusion)	None	Pre op	1	2.5	60	Excellent results/recovery in 78% of patients	104
Eltorai et al., 1984, USA [70]	44	44 M	51	Retrospective cohort	Infectious	SCI patients with chronic osteomyelitis	None	NS	Surgery (debridement/ostectomy/amputation)	Antibiotics	Chronic phase (1–30 years after SCI)	30–70	2.0	120	68% cured, 11% recurrence, 20% failed	26–468

**Table 4:** TSCI case reports/small series.

Author, Year, Country	Age, Sex	Etiology	Initial Treatment	AIS Grade	MRI	Spinal Surgery	Adjunctive Medical Therapy	Start of HBOT	N° of Sessions	ATA	Duration of Each Session (min)	Outcome	Follow-Up (Week)
Zhang et al., 2023, China [71]	38, F	Brown-Séquard syndrome	Neurotrophic drugs (mecobalamin), tizanidine	AIS B	Congenital C2-C5 fusion, stenosis, cord compression	Posterior decompression + C1-C2 fixation with plate-screw	Rehab, acupuncture	During initial hospitalization (timing not detailed)	NS	NS	NS	Improved to ASIA D, partial independence with aids	78
Tang et al., 2023, China [72]	33, M	Forklift accident, with subsequent lumbosacral spondyloptosis with locked L5 articular process	Stabilization, supportive care	ASIA C	Complete rupture L5-S1 disc, dural tear, ligamentous injury	Posterior instrumentation L3-S2, decompression, cage fusion	Rehab	Post-op day 10	NS	NS	NS	Improved to ASIA D; pain reduced; urinary retention improved	24
Marrosu et al., 2021, Italy [73]	45, M	Traumatic C1 lesion after diving accident	Corticosteroids	NS	Non-hemorrhagic edematous lesion, posterior C1	None	Rehab	3 months post injury	127	2.5	60	Improved proprioception, motor performance, and cortical connectivity	60
Ye et al., 2010, China [74]	54, M	Brown-Séquard syndrome	None	NS	Lamina T5 fracture + intraspinal metal fragment	Decompressive laminectomy + fragment removal	Ceftazidime	Post-op day 10	60	NS	NS	Near-complete motor recovery at 1 year	52

**Table 5:** TSCI cohort studies/trials.

Author, Year, Country	N° of Patients (HBOT Cohort)	Sex	Mean Age	Study Type	Etiology	Initial Treatment	AIS Grade	Spinal Surgery	Adjunctive Medical Therapy	Start of HBOT	N° of Sessions	ATA	Duration (min)	Outcome	Follow-Up (Weeks)
Zhang et al., 2022, China [75]	40	M/F ≈ 3:1	43.2 ± 11.5;	Retrospective cohort	Incomplete cervical TSCI	Corticosteroids	AIS B–D	Decompression + fixation	Rehab	Post-op day 90	30	2.0	95	Improvement to AIS C–E	156
Sun et al., 2019, China [76]	41	NS	53.7 ± 11.8	Randomized clinical trial	Acute TSCI	Corticosteroids	AIS A–D	Some received decompression	Mannitol, rehab	Post-op day 3	30	2.0	115	Improvement to AIS C–E	4
Tan et al., 2018, China [77]	29	21 M/8 F	39.5 ± 10.6	Retrospective cohort	Acute TSCI	None	AIS A–D	Early decompression	Corticosteroids, mecobalamin	Post-op day 3	30	2.0	160	86.2% ≥1 AIS improvement to AIS C–E; MRI lesion size decreased	4
Feng et al., 2017, China [78]	20	14 M/6 F	36.1 ± 5.2	Randomized clinical trial	Incomplete TSCI	None	AIS B–D	Early decompression	Rehab	Post-op day 15	48	2.0	110	Improvement to AIS C–E	8
Ishihara et al., 2001, Japan [79]	22	15 M/7 F	50	Prospective cohort	Acute TSCI	None	AIS A–D	9 fusion, 5 laminoplasty	Corticosterone	Post-op day 1–33	1	2.5	60	Early HBOT response correlated with better recovery: most improved to AIS D/E	104–468
Asamoto et al., 2000, Japan [80]	13	10 M/3 F	60.5	Retrospective cohort	Acute cervical hyperextension TSCI	Supportive care	AIS A–C	None	None	Within hours of onset	3–33	2.0	85	Improved of AIS A to AIS B and AIS B/C to E	12–33
Gamache et al., 1981, USA [81]	25	23 M/2 F	24	Prospective cohort	Acute TSCI	Steroids	NS	6 surgical stabilizations, others external	Corticosteroids	Within hours of onset	18–46	2.0–2.5	90–120	4/25 incomplete SCI improved significantly; complete SCI no recovery	24
Jones et al., 1978, Australia [82]	9	NS	NS	Prospective case series	Acute TSCI	Supportive care	NS	1 had decompression laminectomy	None	Within hours of onset	1–2	2.5	120	5/7 improved neurologically, 2 had functional recovery	24
Yeo et al., 1978, Australia [83]	10	NS	NS	Prospective case series	Acute TSCI (8 paraplegic, 2 tetraplegic)	Supportive care	NS	1 had laminectomy	None	Within hours of onset	2	2.5	90	5/10 improved neurologically; 4 remained unchanged; 1 died of sepsis	24

**Table 6:** Comparison between TSCI and NTSCI.

Feature	TSCI	NTSCI
Typical etiology	Acute traumatic injury	Ischemic, decompression sickness, infectious, radiation
Timing of HBOT	Mostly acute (hours–days)	Acute in vascular/DCS; variable in others
Responsive patients	Incomplete lesions (AIS B–D)	Ischemic and DCS > radiation/chronic
HBOT’s role	Adjunct to surgery and steroids	Adjunct to etiology-specific therapy
Main mechanism	Secondary injury attenuation	Reversal of hypoxia and tissue repair
Strength of evidence	Small cohorts and trials	Predominantly retrospective cohorts

## Data Availability

All data are available within the manuscript. Any additional information can be requested from the corresponding author.
